# A Qualitative Analysis of an Aerobic Interval Training Programme for Obese Outpatients Carried Out in a Hospital Context

**DOI:** 10.3390/ijerph17010312

**Published:** 2020-01-02

**Authors:** Esther Cabanillas-Cruz, Christian López-Rodriguez, Cristina Romero-Blanco, Susana Aznar

**Affiliations:** 1 PAFS Research Group. Faculty of Sports Sciences, University of Castilla-La Mancha, 28310 Toledo, Spain; Esther.Cabanillas@uclm.es (E.C.-C.); christianlr2655@hotmail.com (C.L.-R.); 2 PAFS Research Group, Faculty of Nursing, University of Castilla-La Mancha, 13001 Ciudad Real, Spain; Cristina.romero@uclm.es

**Keywords:** obesity, physical exercise, interview, health

## Abstract

*Purpose*: To describe the experience of obese adults following participation in an indoor aerobic interval training (AIT) intervention. *Methodology*: Qualitative, in-depth semi-structured individual interviews, using phenomenology, with 24 obese adults (body mass index (BMI) ≥30 kg/m^2^) from the Endocrinology Department, at the Severo Ochoa Hospital in Leganés (Madrid). Questions were related to: (a) The physical activity (PA) programme, (b) their perspectives of the activity and exercise environment and (c) the perceived benefits from participation. Data were analysed with a constant comparison approach. *Results*: The main patients’ reasons for beginning the programme were motivations to take part including weight loss, health improvement and doctors’ recommendation. Also, patients showed doubts and feelings of apprehension at the beginning of the intervention. Patients highlighted the role of the instructor, feelings of exercising in a safe environment, a good intervention and accessibility of the facility. They reported an improvement in their quality of life and recommended continuing the program. *Conclusions*: (1) Common themes in the qualitative analysis included enjoyment of the activity and a desire to maintain physical fitness, (2) AIT was perceived as a suitable exercise programme for this population and (3) recommendations for further investigation to understand the role of PA programmes for people with obesity.

## 1. Introduction

Levels of physical inactivity are high in most regions of the world. At least 60% of the global population does not take part in sufficient physical activity (PA), which is necessary to obtain health benefits [1]. In Europe, 54% of adults do not meet current European recommendations for PA. Spain is currently the one of the worst European country for levels of inactivity, with almost 67% of the population not meeting guidelines, and furthermore, two out of three people in the Spanish population spend between 2.5 and 6.5 h per day sitting [2,3]. Sedentary behaviour has been associated with a greater risk of cardiovascular disease [4]. Nowadays, it is known that adults who suffer from obesity obtain benefits from PA, and PA has become a key element in national and international health promotion policies [5,6]. Moreover, PA is associated with a lower rate of chronic diseases and premature death.

Current PA recommendations state that people should accumulate 150 min/week of moderate intensity, or 75 min/week of vigorous intensity in sessions of >10 min. Additional health benefits can be obtained by increasing PA to 300 min/week of aerobic activity of moderate intensity or 150 min/week of vigorous intensity. Furthermore, recommendations also suggest that moderate or high intensity muscle strength activities or exercises should be carried out at least 2 days/week [7].

A significant proportion of the population does not achieve these objectives, and for that reason, individual and public health policies have been developed in an attempt to support the increase in levels of PA [8,9,10,11]. There are significant groups in the population where there is known levels of inactivity, higher than national figures suggest. One of these groups is people who are overweight and obese [12]. These groups are described as ‘hard to reach’, as they are less likely to participate in programmes available in the community. As a consequence, there is a lack of research on how adults with obesity experience PA. It is important to explore this theme to be able to inform the design and future direction of exercise programmes for people with obesity [13].

High intensity interval training (HIIT) is an option for promoting greater health benefits for this population. HIIT as a training method offers a notable and efficient strategy for the time invested in promoting health [14,15]. HIIT protocols include SIT (sprint interval training) and AIT (aerobic interval training), where the former has short intervals, such as 30 s, the latter has longer ones, such as 3 min to 4 min. The most common modality using HIIT with populations with chronic pathologies is AIT, which consists of four to five intervals of 4 min duration at an intensity of around 85–90% of peak heart rate (HR_peak_), followed by a recovery period of 3 min at about 70% of HR_peak_ [16,17,18,19,20]. AIT is well-tolerated, safe, improves quality of life and promotes adherence to exercise for overweight/obese patients and overweight/obese patients with cardiovascular disease and/or metabolic syndrome [21,22,23,24,25].

As far as we know, there are very few studies that have explored patients with obesity perceptions of PA programmes as a clinical intervention based in community sports facilities. There is a need to understand patients’ perceptions of these exercise programmes to improve uptake, adherence and longer-term maintenance.

The purpose of the present qualitative study was to explore participants experiences, understandings and perceptions of the AIT programme using indoor cycling. Semi-structured interviews were conducted with 24 obese patients to explore their progress in the development of the training process, considering their experiences, attitudes and perceived benefits in their general state of health and the results of participating in the programme.

## 2. Materials and Methods

A qualitative method using phenomenology [26,27], which is oriented toward the description that people give of their experience, was used. The participants in the study were recruited from the Department of Endocrinology from a hospital based in Madrid. The inclusion criteria were: Adults with obesity (BMI ≥ 30 kg/m^2^) living in the community. Exclusion criteria included: Regular physically active patients, orthopaedic problems, waiting for bariatric surgery, uncontrolled hypertension, ischemic cardiac, cerebrovascular disease, advanced metabolic disease and/or uncontrolled pulmonary disease. After administering the inclusion and exclusion criteria, the cohort was reduced to 24 participants (16 women and eight men, mean age: 52.8 ± 15.7 years) who were willing to participate in the AIT programme. As part of the intervention, participants also attended healthy eating workshops and psychological support meetings monthly, which were organised by the hospital. Table 1 provides descriptive characteristics of the sample (age, sex, diagnosis and related complementary treatment).

### 2.1. Data Collection

For recruitment, the first author contacted patients in a group meeting at the hospital to explain the project, to inform them about the objectives of the study and to ask for their consent to participate. Following recruitment, patients were contacted to arrange appointments for the interviews. Written informed consent was obtained from each participant before beginning the interview and confidentiality was assured. Using semi-structured interview guided by a phenomenology approach [28], a set of open questions was used to guide discussions. Interviews lasted approximately 45 min, until data saturation and an understanding of the meaning of the replies were achieved. The interviews were carried out in a private small room at the sports hall in an atmosphere of trust and cordiality, which permitted the participants to freely express their opinions. All interviews were administered in the morning between 11:00 h and 13:00 h. The interviews were recorded and transcribed verbatim. The transcriptions were given back to the participants for member checking. The researcher, a clinical psychologist specialized in qualitative methods, was familiar with the context. The transcription data were analysed using thematic content analysis [29].

### 2.2. Ethical Considerations

This research complied with the stipulations of the Declaration of Helsinki and the legislation on protection of personal data. The study protocol and the methodology were approved by the Ethics Committee for Clinical Research at the Hospital where it was undertaken (Registered number: 549-A (28/11), Leganés, Madrid)). The participants were informed of the study procedures and protocols in two monthly meetings organized by the Hospital.

This research did not receive any specific subsidy from the financing organs of the public sector, or commercial or non-profit organisms.

### 2.3. Training Protocol

**Figure 1:**
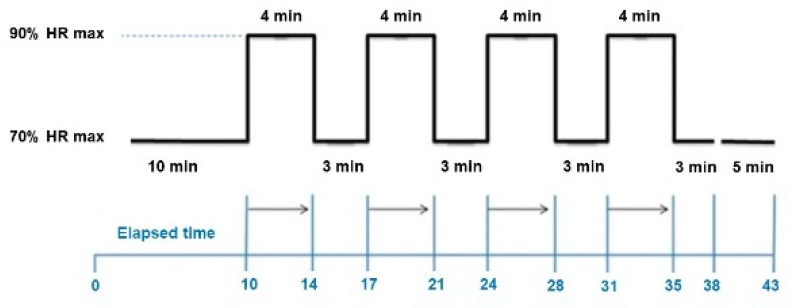
Training protocol.

Patients participated in 52 exercise sessions. Sessions were carried out three times per week for five months. The AIT training used indoor cycling for the exercise mode. Each training session started with a 10-min warm up at 70% of HR_peak_ followed by four intervals of 4 min each at 90% HR_peak_. Between each interval, there was an active recovery period of 3 min at 70% of HR_peak_. (see Figure 1). The cooldown consisted of 5 min of cycling slowly and some stretching exercises while still sitting on the bike [16]. In terms of adherence, all patients completed 100% of the programme.

### 2.4. Data Analysis

The analysis followed the steps described for a phenomenology study [28]. Initially, the research team read and reread the transcribed data several times to familiarize themselves with the content. In the next step, the different statements were identified and grouped into themes and sub-themes according to their previous understanding, based on similarities and differences. Finally, different descriptive categories were created, representing the main themes of interest identified by the research team. The research team was involved throughout the process, and frequent meetings were held [30].

## 3. Results

Table 2 presents the themes and their subthemes. They are presented in the text with selected quotes to provide the lived experience of the participants in the findings.

Three themes emerged from the analysis: Theme 1 includes the first four subthemes on general aspects of the physical exercise programme; Theme 2 integrates subthemes 5 and 6, alluding to the perspective on the activity and the environment; and Theme 3 consists of the subthemes 7 and 8, with reference to the improvements observed, and other aspects which contribute to the improvement of the programme. Each theme was analysed specifically.

### 3.1. Theme 1: General Aspects of the Physical Exercise Programme

The participants’ perceptions deal with the following themes: Their opinion of the programme, the way they heard about it, the reasons for beginning it and the demand of the obese patients. The patients’ perspectives represent a global vision of the whole intervention programme.

Subtheme 1: *Opinions of the programme*. Patients explained the programme according to their own experiences or beliefs, and the patients gave a positive opinion of the physical exercise programme. This opinion basically referred to the improvements that they observed in their lives. There are patients who defined the programme as beneficial for them, and considered that it was also beneficial for their close relatives and other people.

Subtheme 2: *The way they heard about the programme*. The way that they heard about the existence of the programme is an important fact for discovering its scope. Findings showed that the majority heard about it from health professionals (doctors, nurses, physiotherapists and psychologists) and from the recommendations of others who were already practising it. The patients stated that before the referral to the programme, they did not know about it.

Subtheme 3: *Reasons for starting the programme*. There were three reasons why the patients decided to participate in the physical exercise programme: To lose weight, to improve their health and because of the doctor’s recommendation. Other patients stated that their reason for starting was due to interest from their family.

Subtheme 4. *Expectations.* There were three aspects: One was the doubts that they referred to in their replies. With regard to their sensations, their opinion involved need to improve their health status. As well as considering that they felt somewhat lazy about beginning the programme, they added that it was an activity that would do them good. Last, they stated that a physical exercise programme was necessary, but that there was a certain amount of apprehension about following it.

### 3.2. Theme 2. Perspective on the Activity and the Environment

The findings revealed that a favourable or unfavourable perspective of the physical exercise programme can be due to different aspects of the environment. As the most important aspects, patients highlighted the instructor, accessibility, the facilities and the safety of the activity, reflecting the negative experiences that they may have had during a session.

Subtheme 5: *The instructor*. Regarding their perspective on the instructor in the physical exercise programme, patients highlighted different qualities that he should possess to do the work well and manage to positively influence the participants taking their disease into account, including motivation, empathy and, similarly, enthusiasm. The patients who mentioned motivation referred to the capacity of the instructor to positively influence their states of mind to achieve their goals. For them, empathy was understood as affection and warmth, both with the individual and the group, and enthusiasm as his own enjoyment of the activity.

Subtheme 6: *Activity and environment*. This perspective refers to the environment where the activity took place and the programme characteristics, such as accessibility, facilities and timetable. The three aspects were often mentioned by the patients who alleged different opinions, both favourable and unfavourable. Moreover, safety was another fundamental aspect that they highlighted. The degree of accessibility, classified as good, seemed to be the principal concern of the participants, who mainly asserted that the facility was in the centre of the town. A few patients commented on unfavourable aspects, like inadequate equipment which could be improved, but the equipment was suitable for the classes. Another weakness pointed out was the timetable, and the need for a more flexible timetable for future interventions was often mentioned. Last, safety was a facet which was related to the existence of some negative experience during the course of the programme. In this respect, the patients answered emphatically that they had never had any mishaps.

### 3.3. Perceived Benefits

This category was related to two subthemes: Benefits and recommendations. Benefits included the improvements observed and their repercussions in everyday life, while the recommendations included those that the patients would make for other people to begin a physical exercise programme.

Subtheme 7: *Overall benefits*. This theme corresponds to physical fitness, positive mood state and socioaffective improvements and are difficult for others to observe, as well as the repercussion of the mentioned improvements on their daily lives. At the physical level, the patients mainly indicated improvements in cardiorespiratory fitness (felt less tired), and in strength and increase muscle tone, as well as other improvements in the psychological context. The patients also underlined their improvements in coordination, relating this to agility. At the socioaffective level, they highlighted a more pronounced manifestation of their emotions, which they related to their mood and expressions of happiness or enjoyment when they carried out the training sessions. Moreover, they highlighted improvements in expression and socialization, alluding to questions like communicative intent, as well as the fact that they related better to their environment, creating a group climate suitable for following a physical exercise programme. Few patients referred to the cognitive level, that is, alluding to the learning that they acquired in the training sessions to be able to implement them independently at the end of the intervention. With regard to the repercussions of the improvements in their daily lives, this perception was produced by an enhancement of their quality of life thanks to the physical fitness and socioaffective improvements, which generated more independence in the individual. Last, they referred to improvements that were not easy to observe because they developed very slowly and were difficult to measure.

In fact, not as part of this study, subjects showed physiological improvements in cardiorespiratory fitness, decreased blood pressure and decreased % body fat, which can corroborate these qualitative results (see supplementary material Table S1).

Subtheme 8: *Future recommendations*. From this perspective, the patients recommended this physical exercise programme based both on their experience, and on the improvements seen in others, also highlighting the enjoyment of the activity itself. Moreover, patients stated their desire to continue with the proposed intervention programme.

## 4. Discussion

Obese individuals are encouraged to participate in PA. However, there are few qualitative studies that explored the motivations and experiences of obese individuals regarding PA. Thus, this study may be useful for the future direction of programme with this client group and complements previous research in the area using qualitative methods [30,31]. The results showed that despite the patients beginning the exercise programme to lose weight, improve their health and join the programme through their doctor’s recommendation, they experienced doubts and feelings of apprehension about starting the programme. The patients highlighted the key role of the instructor, the safety of the activity and the accessibility of the facility. They perceived socioaffecive and psychomotor improvements which translated into an improvement in their satisfaction with care, and as a consequence, recommended that the intervention programme should continue with the possibility of including a larger number of patients.

The use of AIT for the exercise training protocol may have influenced the adherence to the programme. The positive benefits of enjoyment, short exercise protocol when compared to moderate continuous exercise protocols are particularly important for sedentary people and patients [32] Moreover, HIIT protocols have been encouraged for obtaining similar fat loss [33] or more absolute fat loss [34] when compared to moderate intensity continuous exercise protocols.

Some of the study themes concur with the findings of other researchers. Doing exercise with someone who has the same level of fitness increases motivation [35]. PA is experienced positively by adults with obesity, but there are many obstacles that influence their will and capacity [36]. Support was necessary in different ways and contributed to not only beginning the PA, but also to keeping it up. The enjoyment of the activity was often a theme mentioned by the patients. For continued commitment, it was very important to find an environment where they felt safe, accepted and encouraged to be active. In other words, social support positively influenced their motivation, self-efficacy and goal setting. On the other hand, social and environmental factors, like lack of accessibility to opportunities for PA, predicted dropping out.

Perceived self-efficacy, or the belief in one’s own capabilities to achieve a behavioural objective, are fundamental [36,37]. This may be related with the search for meaning, which is not a product of culture, but emanates from the deepest part of the human being, as a primary necessity, which may remain dormant but which in determined contexts, forcefully emerges. The motives for participating in the PA were diverse, the themes that emerged were health, pressure, pleasure and feelings of achievement. Here, the recommendations of the health professionals were prominent in their hearing of the exercise programme and their reasons for beginning it. In spite of the health benefits of increasing PA little by little, physicians were cautious when it was a question of integrating PA into the health paradigm. Health professionals should take on more responsibility to influence their patients and the population in general to be more physically active [38]. High self-motivation, determination and persistence pursuing determined objectives are positively associated with exercise adherence [39]. The participants reasons reflected several degrees of perceived self-determination, which ranged from very little (“*because those are the rules; I have to do it*”), to scant self-determination (“*because I would feel guilty if I didn’t do it*”) and a lot of determination (“*because it is important for me*”), to showing total self-determination (“*because it is fun and I enjoy it*”). The more the participants’ motivations were self-determined, the more effort they put into the programme and the more they enjoyed achieving new physical goals.

When people seek to change their behaviour, they are often inspired by guidance of an internal (goals) or external (rules) nature to do it. For example, to lose weight, they can be inspired by internal guidance (“*I think that it is the best way to help myself*”) or external guidance (“*I want others to see that I really am trying to lose weight*”). However, many obese people could become frustrated with exercise when their efforts do not lead to the corresponding changes in weight or body composition. Instead of promoting weight loss in obese people, it is more suitable and probably more effective to encourage them to become more physically active, that is, create learning so that the obese patients continue independently after the intervention programme finishes. Although they were shown the necessary tools to extrapolate them to their daily lives, it would nonetheless be advisable in future programmes to use other strategies to achieve these ends. It has been shown that the participants who become physically more active and have a moderate level of fitness are more likely to continue doing exercise [40].

These mediators of exercise adherence were taken into account at the beginning of the training programme, highlighting the key role played by the instructor in the achievement of objectives, his commitment during the whole programme and the creation of a group climate suitable for this interval training activity. According to the patients in the study, the instructor should possess qualities like motivation, empathy and enthusiasm for an activity to be successful and the proposed objectives achieved. Thus, it is proposed that the instructor develop leadership based on coaching [41], which is characterised by discipline through values and personal example, with concrete objectives for each individual and the group. The instructor maintains, during the whole programme, a proactive and flexible attitude using individualised motivational systems, carrying out daily adjustments and adapting immediately to the circumstances that arise.

The results in the present study provide a good support for our analysis of the multiple mechanisms by which past behaviour can affect future responses. Past behaviour contributes directly to future endeavours in contexts that support the development of habits. Behaviours that are practised well and carried out in stable contexts will probably be repeated. Generally speaking, human behaviour can be predicted according to social and psychological factors. With respect to psychological factors, we can differentiate between affective (moods and emotions) and cognitive (beliefs and expectations) characteristics. Social factors (social networks and norms) act to facilitate or inhibit the manifestation of a given behaviour. All the mentioned factors should be considered when predicting the appearance, maintenance or extinction of a behaviour in a determined context [42].

### Limitations

All the factors presented in this study were highly specific to the local context and the particular exercise programme (AIT) which, together with the use of a specifically recruited sample, made the results difficult to generalize. The ideal scenario would be that the study was extended to include more medical institutions and examine patients of different levels of obesity and demographic profiles. Furthermore, the experiences of the patients obtained in this study come from different participants and could have been affected by their personal characteristics. As such, the study recommends that future research is carried out either as a longitudinal study on the same group of patients or with more groups in a variety of settings. Moreover, positive results in some physiological outcomes can affect the psychological response, and therefore future research should take psychological factors into consideration (i.e., analysing data according to getting or not getting the expected benefits).

## 5. Conclusions

The study presents three key conclusions: (1) Common themes in the qualitative analysis included enjoyment of the activity and a desire to maintain physical fitness; (2) AIT was perceived as a suitable exercise programme for this population and (3) recommendations for further investigations to understand the role of PA programmes for people with obesity.

## Figures and Tables

**Table 1:** Descriptive characteristics of the sample.

Participant Nº	Age	Sex (F/M)	Diagnosis ^a^	Other therapies (Diet/Exercise) ^b^
1	72	F	Obesity and hypertension ^a^	Not following hospital hypocaloric diet
2	53	M	Obesity	Following hospital hypocaloric diet
3	67	F	Obesity	Following hospital hypocaloric diet
4	35	F	Obesity	Following hospital hypocaloric diet
5	45	M	Obesity	Following hospital hypocaloric diet and swimming 2 times/week
6	75	M	Obesity	Following hospital hypocaloric diet
7	39	F	Obesity and hypertension	Following hospital hypocaloric diet
8	46	F	Obesity	Not following hospital hypocaloric diet
9	40	F	Obesity	Not following hospital hypocaloric diet
10	40	M	Obesity and hypertension	Not following hospital hypocaloric diet
11	34	M	Obesity and hypertension	Following hospital hypocaloric diet
12	69	M	Obesity and hypertension	Following hospital hypocaloric diet
13	52	F	Obesity and hypertension	Not following hospital hypocaloric diet
14	61	F	Obesity	Following hospital hypocaloric diet
15	43	F	Obesity and hypertension	Following hospital hypocaloric diet
16	74	F	Obesity and type 2 diabetes	Following hospital hypocaloric diet
17	65	M	Obesity	Not following hospital hypocaloric diet and cycling 2 times/week
18	54	F	Obesity	Following hospital hypocaloric diet
19	41	F	Obesity	Following hospital hypocaloric diet
20	59	F	Obesity and hypertension	Following hospital hypocaloric diet
21	45	F	Obesity	Not following hospital hypocaloric diet
22	57	M	Obesity	Following hospital hypocaloric diet
23	48	F	Obesity	Following hospital hypocaloric diet
24	53	F	Obesity	Following hospital hypocaloric diet

^a^ Obesity is defined as a body mass index ≥30 kg/m^2^; hypertension is defined by values of systolic pressure ≥140 mmHg or diastolic pressure ≥90 mmHg; type 2 diabetes is defined as a plasma glucose concentration ≥126 mg/dL or ≥200 mg/dL 2 h after drinking a solution with 75 g of glucose. ^b^ A diet calculated according to the weight of each patient on their first visit to the Unit of Endocrinology at the hospital, multiplied by 20 kcal, in a range that can oscillate between 1500–2000 calories. Monthly controls were carried out in the eating workshops organized by the hospital.

**Table 2:** Summary of findings.

Theme	Subtheme	Quotes ^a^
General aspects of the programme	1. Opinions of the programme	‘...*I liked it, I feel more encouraged and I am better physically*’ (20)
	2. How patient received information on the programme	‘*My doctor recommended it*’ (15) ‘*Because my daughter participated in a programme like this and I saw the improvement, also to motivate and encourage myself*’ (13)
	3. Reasons for starting the programme	‘*To get slimmer*’ (2) ‘*Because I felt bad physically and emotionally*’ (4)
	4.Expectations	‘*It was time to do something*’ (9) ‘*I thought it would be very good for me*’ (15) ‘*Fear to be able to do it”* (17)
Opinion of the activity and the instructor	5. Instructor	‘*The people themselves, especially the instructor, who has motivated us with his enthusiasm as we only had to compete against ourselves*’ (13)
	6. Activity and environment	‘*Fantastic, very at ease*’ (2) ‘*I live a long way from the facility.*’ (15) ‘*Better equipment for the bikes*’ (10) ‘*Being sure that the programme was suitable*’ (22)
Perceived benefits	7. Overall benefits	*‘Stronger, better psychologically*’ (16) ‘*I feel better, more agile. Before I thought that I couldn’t do it*’ (8) ‘*I have learned a lot in a short time, I am grateful, I must continue so that I don’t put on weight*’ (17) ‘*Surprised by my participation and constancy, improved mood, etc.*’ (20)
	8. Future recommendations	*‘Excellent, the programme should continue*’ (3) ‘*Recommendable for more people*’ (11) ‘*It is fantastic and very complete*’ (23) ‘*It should be repeated*’ (6)

^a^ Quotes are presented for each theme. The number in parenthesis identifies the interview where the quote comes from.

## Supplementary Materials

The following are available online at https://www.mdpi.com/1660-4601/17/1/312/s1, Table S1: Supplementary material.
