# Supplementary material for: A Qualitative Analysis of an Aerobic Interval Training Programme for Obese Outpatients Carried Out in a Hospital Context
Source: Int J Environ Res Public Health. 2020 Jan 2;17(1):312. doi: 10.3390/ijerph17010312 (PMC6981676; doi:10.3390/ijerph17010312)
Supplement: Supplementary file 1 [file ijerph-17-00312-s001.pdf]

Supplementary material

Table S1. Supplementary material.

	Control Group			AIT Group			*p* (Inter-Groups)	
	**Pretest**	**Posttest**	***p* (Intra-Groups)**	**Pretest**	**Posttest**	***p* (Intra-Groups)**	**Pretest**	**Posttest**
**Body Composition**								
Body weight (kg)	103.55 ± 21.98	103.58 ± 22.35	0.954	94.47 ± 11.84	93.25 ± 10.79	0.078	0.083	0.050
Waist circumference (cm)	116.68 ± 13.39	119.52 ± 17.40	0.223	107.75 ± 8.98	100.06 ± 20.47	0.092	0.009 **	0.001 **
BMI (kg/m^2)	38.16 ± 6.66	38.40 ± 6.78	0.227	35.65 ± 3.34	35.32 ± 3.40	0.218	0.108	0.055
Body fat (%)	44.04 ± 7.58	43.71 ± 7.70	0.058	42.60 ± 6.23	41.68 ± 6.68	0.014 *	0.478	0.334
Muscle Mass (%)	56.02 ± 7.58	56.20 ± 7.76	0.255	56.33 ± 5.40	57.17 ± 5.92	0.441	0.872	0.628
**Blood Pressure**								
SBP (mmHg)	146.22 ± 14.92	146.11 ± 17.28	0.965	134.35 ± 15.55	123.29 ± 7.75	0.005 **	0.073	0.000 **
DBP (mmHg)	80.78 ± 11.16	81.89 ± 8.12	0.575	79.76 ± 8.16	74.06 ± 6.93	0.009 **	0.793	0.016 *
**CF**								
VO_{2peak} (ml/kg/min)	14.05 ± 2.01	13.83 ± 2.11	0.411	17.58 ± 4.30	20.07 ± 4.49	0.000 **	0.001 **	0.000 **
FC_{peak} (b/min)	137.83 ± 14.11	136.96 ± 12.11	0.700	152.38 ± 23.78	145.04 ± 21.13	0.021 *	0.014 *	0.113
Workload peak (W)	95.20 ± 26.81	85.62 ± 26.92	0.002 **	128.12 ± 41.72	145.00 ± 46.20	0.000 **	0.002 **	0.000 **
VO_2 at VT (ml/kg/min)	9.98 ± 0.82	10.27 ± 1.61	0.338	12.03 ± 2.97	13.56 ± 3.68	0.001 **	0.003 **	0.000 **
**Blood variables**								
Cholesterol (mg/dl)	171.00 ± 33.64	165.63 ± 25.87	0.064	186.88 ± 27.50	186.50 ± 28.96	0.901	0.080	0.011 *
Cholesterol HDL (mg/dl)	47.75 ± 11.90	49.75 ± 12.80	0.025 *	51.17 ± 11.57	53.58 ± 10.84	0.070	0.318	0.269
Triglycerides (mg/dl)	127.13 ± 53.36	123.00 ± 56.62	0.547	108.33 ± 42.49	101.17 ± 43.03	0.113	0.184	0.140
Glucose (mg/dl)	107.17 ± 25.45	108.54 ± 23.71	0.410	101.88 ± 14.94	103.00 ± 16.23	0.648	0.384	0.350
HOMA	4.62 ± 2.57	5.69 ± 2.94	0.002 **	3.78 ± 2.08	2.61 ± 1.10	0.001 **	0.215	0.000 **

* $p < 0.1$, ** $p < 0.05$.
